# Development and Application of a Novel Rapid and Throughput Method for Broad-Spectrum Anti-Foodborne Norovirus Antibody Testing

**DOI:** 10.3389/fmicb.2021.670488

**Published:** 2021-09-03

**Authors:** Yueting Zuo, Liang Xue, Junshan Gao, Yingyin Liao, Yueting Jiang, Ying Li, Yanhui Liang, Linping Wang, Weicheng Cai, Tong Cheng, Juan Wang, Moutong Chen, Jumei Zhang, Yu Ding, Qingping Wu

**Affiliations:** ^1^School of Bioscience and Bioengineering, South China University of Technology, Guangzhou, China; ^2^Guangdong Provincial Key Laboratory of Microbial Safety and Health, State Key Laboratory of Applied Microbiology Southern China, Institute of Microbiology, Guangdong Academy of Sciences, Guangzhou, China; ^3^Department of Laboratory Medicine, First Affiliated Hospital of Guangzhou Medical University, Guangzhou, China

**Keywords:** norovirus, capsid P protein, enzyme-linked immunosorbent assay, broad-spectrum antibodies, inter-assay/intra-assay variation

## Abstract

Foodbone norovirus (NoV) is the leading cause of acute gastroenteritis worldwide. Candidate vaccines are being developed, however, no licensed vaccines are currently available for managing NoV infections. Screening for stimulated antibodies with broad-spectrum binding activities can be performed for the development of NoV polyvalent vaccines. In this study, we aimed to develop an indirect enzyme-linked immunosorbent assay (ELISA) for testing the broad spectrum of anti-NoV antibodies. Capsid P proteins from 28 representative NoV strains (GI.1–GI.9 and GII.1–GII.22 except GII.11, GII.18, and GII.19) were selected, prepared, and used as coating antigens on one microplate. Combined with incubation and the horseradish peroxidase chromogenic reaction, the entire process for testing the spectrum of unknown antibodies required 2 h for completion. The intra-assay and inter-assay coefficients of variation were less than 10%. The new method was successfully performed with monoclonal antibodies and polyclonal antibodies induced by multiple antigens. In conclusion, the indirect ELISA assay developed in this study had a good performance of reliability, convenience, and high-throughput screening for broad-spectrum antibodies.

## Introduction

Foodbone Norovirus (NoV) is one of the leading causes of epidemic acute gastroenteritis (AGE) worldwide, accounting for one-fifth of all gastroenteritis cases globally (Lopman et al., 2016). Annually, NoV has been estimated to cause 677 million episodes of diarrheal disease (95% uncertainty interval [UI]: 468–1,153 million) and 213,515 deaths (95% UI: 171,783–266,561) for all ages and among all modes of transmission (Pires et al., 2015). NoV is extremely contagious, and transmission occurs directly from person to person via fecal-oral and vomit-oral routes, but can also be caused by food-borne, water-borne, or environmental factors (Verhoef et al., 2015). Vomitus and feces of infected patients contain a considerable number of virions, whereas as few as 10 infectious particles are sufficient to cause AGE (Teunis et al., 2010). Additionally, NoV shedding after infection usually lasts for several weeks, and prolonged shedding post-infection can also contribute to spreading (Sukhrie et al., 2010). Thus, NoV remains a major threat to public health. Owing to the significant social and economic burden associated with the disease, adequate preventive measures against this virus should be formulated.

NoV strains can be classified into 10 genogroups (GI–GX) and at least 48 genotypes based on the diversity of the VP1 amino acid sequence; GI, GII, and GIV have been shown to cause infections in humans (Chhabra et al., 2019). Human histo-blood group antigens (HBGAs) can be used as receptor or co-receptor for NoV infection (Lindesmith et al., 2003; Rockx et al., 2005). The binding ability between NoVs and HBGAs is diverse, resulting in different infectivity. The global pandemic GII.4 NoV can infect almost all secretory individuals (Nordgren and Svensson, 2019); GI.1 VLP binds to A, AB, and O type, but not to B type saliva (Morozov et al., 2018); individuals with O type saliva are relatively susceptible to NoV, while with B type are not (Hutson et al., 2002); some strains do not bind any phenotype of HGBAs (Almand et al., 2017).

Several possible barriers hinder the development of effective NoV vaccines (Cortes-Penfield et al., 2017). First, circulating NoV exhibits genetic diversity and high variation, which may limit the durability of protection conferred by a vaccine that does not elicit broadly neutralizing antibodies. Second, the lack of sufficient cell lines for virus culture and successful animal models further hinders the development of antiviral drugs and vaccines. The following three types of NoV vaccines have been developed: virus-like particles (VLPs) that resemble the organization and morphology of native virus, P particles that resemble the P domain of native virus, and recombinant adenoviruses (Esposito and Principi, 2020).

Enzyme-linked immunosorbent assay (ELISA) is a sensitive and specific analytical biochemistry assay utilized for the detection and quantitative or qualitative analysis of an analyte without the requirement for sophisticated or expensive equipment (Gan and Patel, 2013). Currently, ELISA is used as an ideal method for the detection of viruses and antibodies (Fan et al., 2013; Ledesma et al., 2017; Ding et al., 2019). However, few ELISA techniques demonstrate the ability to directly detect the broad-spectrum binding activities of antibodies against all NoV strains that infect humans. Therefore, it is necessary to develop an indirect ELISA technique for the detection of a wide range of antibodies.

In this study, we aimed to develop an ELISA technique for vaccine development via rapid, efficient, and accurate screening of a substantial number of broad-spectrum antibodies.

## Materials and Methods

### Selection of Representative Strains

The antigen used in this experiment was capsid P protein for each representative strain of NoV stored in our laboratory. We selected nine GI strains (GI.1–GI.9) and nineteen GII strains (GII.1–GII.22) as representative NoV strains; GII.11, GII.18, and GII.19, which were detected in swine (Vinjé, 2015), were excluded. In our previous study, GII.2, GII.3, GII.4, GII.6, GII.8, and GII.17 NoV were detected and preserved (Xue et al., 2015; Xue et al., 2016; Xue et al., 2019), which could be directly used for cloning. Others were synthesized.

### Pre-coated of Antigen

For preparation of microplates, 96-well plates were coated with 0.2 μg of 28 NoV capsid P proteins after incubation at 4°C overnight (three well per P protein). After subjecting the wells to washing steps with phosphate-buffered saline containing Tween 20 (PBST) three times, the wells were blocked using 5% skim milk for 2 h at 37°C; then, the wells were washed three times using PBST, dried, and stored at 4°C until further use. A combination of glutathione S-transferase (GST)-tagged protein and commercial anti-GST antibodies was used as a quality control. The negative serum was used as a negative control.

### Enzyme Linked Immunosorbent Assay (ELISA)

**FIGURE 1:**
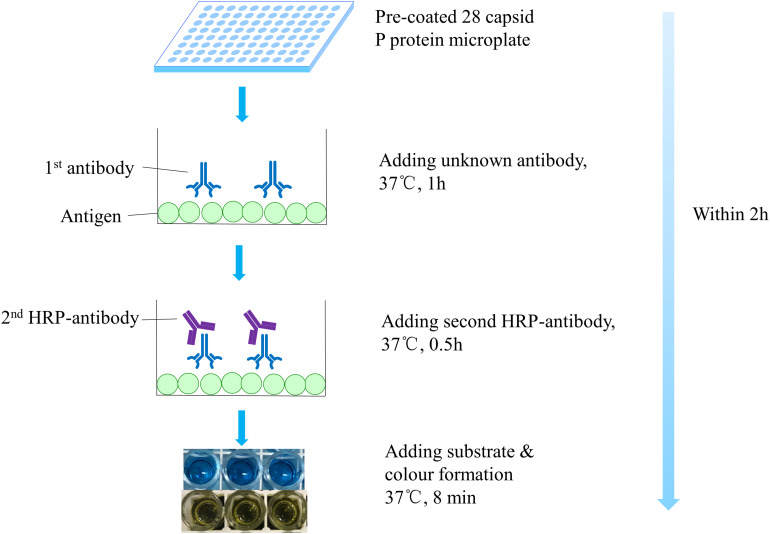
Step-by-step schematic representation of ELISA. Before addition of the antibody to be tested, the dilution corresponding to the *OD* value of 0.8–1.2 was determined. Broad-spectrum norovirus antibodies were determined within 2 h.

The indirect ELISA method was used to detect the broad spectrum of antibodies (Figure 1). Briefly, the pre-coated microtiter plate was taken out. Serum samples were added at an appropriate dilution, which corresponded to the *OD* value in the range of 0.8–1.2, followed by incubation for 1 h at 37°C. Subsequently, secondary horseradish peroxidase-conjugated goat anti-mouse IgG antibodies (diluted 1: 3,000) were added and the plates were incubated for 30 min at 37°C. Tetramethylbenzidine was added as a peroxidase chromogenic substrate and reaction for 8 min at 37°C was performed. After terminating the substrate reaction with 2 M H_2_SO_4_, the optical density (*OD*) was measured at 450 nm. Positive signal is defined as a mean *OD*_450_ >0.2 after background subtraction (Uusi-Kerttula et al., 2014; Jin et al., 2016; Tamminen et al., 2019).

## Performance Analysis

### Intra-Assay Precision

Three replicates for each monoclonal antibody sample were analyzed using the same microplate. Precision was determined by calculating the mean, standard deviation, and coefficient of variation (CV%).

### Inter-Assay Precision

For determination of inter-assay reproducibility, three replicates for each sample were analyzed in different microplates. Precision was determined by calculating the mean, standard deviation, and CV%.

### Statistical Analysis

Statistical analyses were performed using Microsoft Excel 2016 and GraphPad Prism 8.0 software. Broad spectrum plot data are presented as mean ± standard deviation.

## Results

### Assembly of Microplates

**FIGURE 2:**
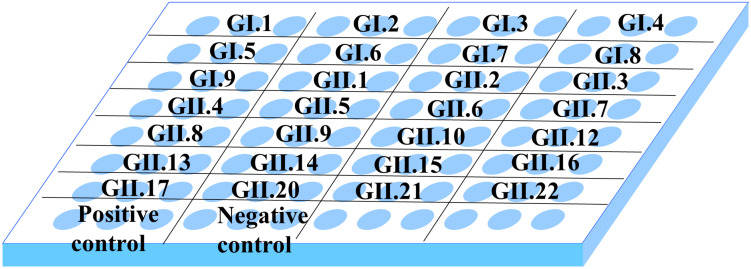
Distribution of antigen coating in 96-well microplate. In the plate, each well was coated with 0.2 μg P protein, and each P protein was coated with three multiple wells. Wells coated with GST and coating buffer served as positive control and negative control, respectively.

To perform this experiment, we selected representative strains for capsid P proteins of all genotypes in GI (GI.1–GI.9) and GII (GII.1–GII.22), except for GII.18, GII.19, and GII.20 (Table 1 and Supplementary Figure 1). As shown in Figure 2, the microplate was coated with 28 capsid P proteins obtained from representative strains, and each P protein was coated in three wells.

**TABLE 1:** Selection of norovirus strains used as coating antigens.

Strain designation	GenBank accession no.
GI.1	NC_001959
GI.2	L07418
GI.3	KJ196292
GI.4	AB042808
GI.5	AB039774
GI.6	LC342057
GI.7	KU311161
GI.8	KJ196298
GI.9	KF586507
GII.1	HCU07611
GII.2	MK729086
GII.3	KY348697
GII.4	KT202793
GII.5	KJ196288
GII.6	JX989075
GII.7	KJ196295
GII.8	MK213549
GII.9	AY038599
GII.10	AF504671
GII.12	AB045603
GII.13	KJ196276
GII.14	KJ196278
GII.15	KJ196290
GII.16	AY772730
GII.17	KT970369
GII.20	EU424333
GII.21	KJ196284
GII.22	MG495082

### Broad-Spectrum Antibody Assay

To determine the effectiveness of the method, two GII.4 monoclonal antibodies (GII.4-4A21C1 and GII.4-5D41A12) and one bivalent (GII.4 + GII.8) polyclonal antibody were tested for broad-spectrum detection. When the *OD* was in the range of 0.8–1.2, the dilution of the above three antibodies were between 1:4000–1:16000, 1:4000–1:16000, and 1:500–1:1000, respectively (unpublished data). Accordingly, the two monoclonal antibodies dilution of 1:5000 and the polyclonal antibody dilution of 1:500 were used for broad-spectrum detection.

**FIGURE 3:**
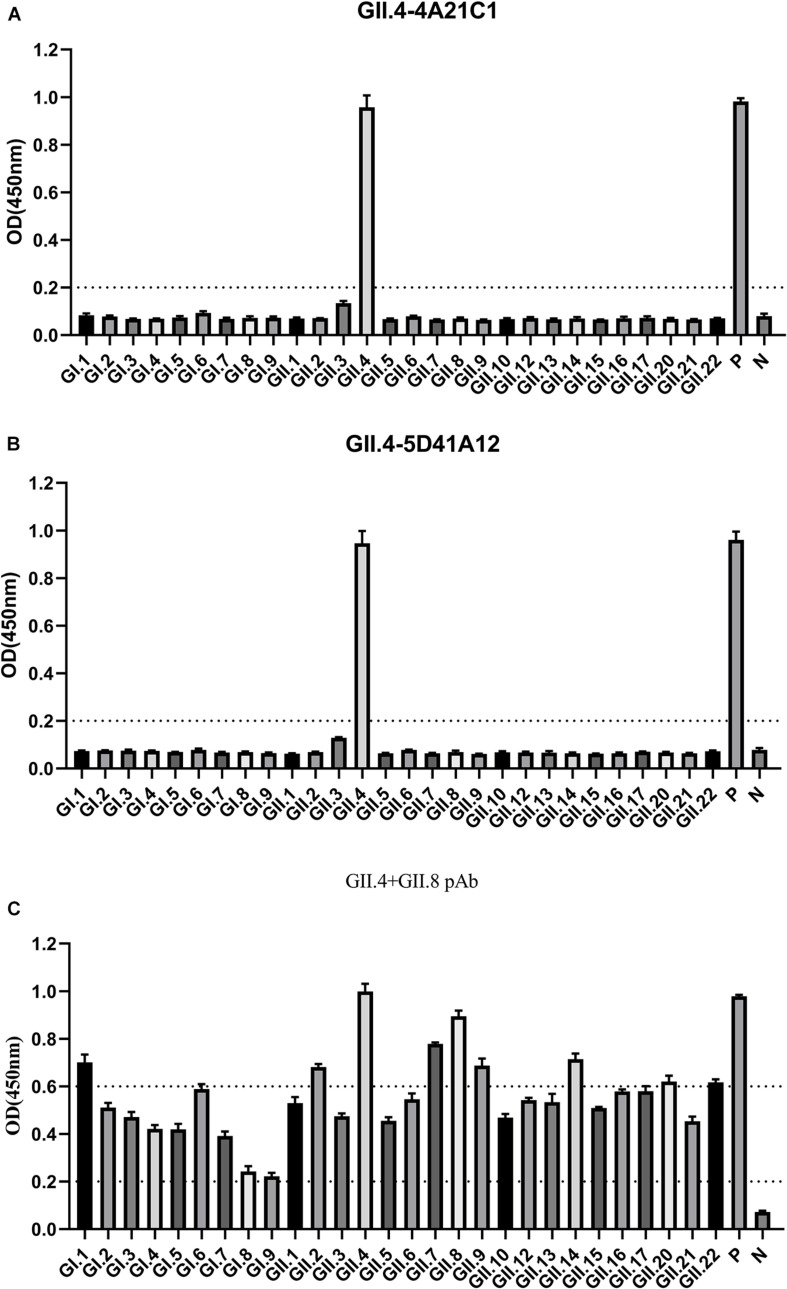
Characterization the broad spectrum of antibodies. 9 GI and 19 GII P proteins were used as antigens with enzyme-linked immunosorbent assay (ELISA), reacting with GII.4-4A21C1 **(A)**, GII.4-5D41A12 **(B)** mAbs, and GII.4 + GII.8 pAbs **(C)**, and each protein was coated with three wells. The horizontal line at 0.2 illustrates the cut-off value for samples that are considered positive. 0.2–0.6 *OD* is considered a moderate binding. *OD* >0.6 represents robust binding. P, positive control; N, negative control.

The GII.4-4A21C1 monoclonal antibodies only showed reaction with GII.4 P particles at a high *OD* value of 0.96 and did not exhibit reaction with the other 27 NoV P particles (Figure 3A), demonstrating that this monoclonal antibody had strong specificity. Similarly, GII.4-5D41A12 monoclonal antibodies had a binding signal of 0.95 (Figure 3B). The polyclonal antibodies (GII.4 + GII.8) showed the highest signal reaction with GII.4 and GII.8 P particles, followed by GI.1, GII.2, GII.7, GII.9, GII.14, GII.20, and GII.22, and moderate signals were observed for the remaining antibodies (Figure 3C). GST bound with GST monoclonal antibodies at *OD* values of 0.98, 0.96, and 0.98, indicating that the developed ELISA technique was reliable.

### Reproducibility of ELISA

Next, we evaluated intra-assay and inter-assay variabilities to test the reproducibility of the method. The monoclonal antibodies GII.4-4A21C1 and GII.4-5D41A12 and the polyclonal antibodies GII.4 + GII.8 were analyzed three times in each microplate. Intra-assay CVs were observed to be in the ranges of 0.63–9.62% (Table 2), 0–9.99% (Table 3), and 0.55–9.88% (Table 4), respectively. After repeating the experiments in three batches of plates prepared for 3 days, the inter-assay CVs were found to be in the ranges of 0.22–9.08% (Table 2), 0.3–9.76% (Table 3), and 0.21–9.99% (Table 4). These results indicated that the developed ELISA technique showed good reproducibility.

**TABLE 2:** Intra- and inter-assay coefficients of variation (%) for the broad-spectrum detection of GII.4-4A21C1 monoclonal antibodies by ELISA.

GII.4-4A21C1	Intra-assay Precision CV/%			Inter-assay Precision CV/%
Antigen designation	1	2	3	–
GI.1	1.80	8.02	9.01	3.52
GI.2	3.87	4.02	5.76	1.83
GI.3	9.45	3.22	2.94	5.37
GI.4	2.12	0.83	2.22	1.65
GI.5	3.81	4.68	7.88	1.98
GI.6	1.67	5.08	7.53	1.43
GI.7	2.50	1.85	7.22	1.08
GI.8	5.65	2.66	8.41	1.52
GI.9	2.55	3.77	6.42	5.26
GII.1	2.59	3.13	5.80	4.07
GII.2	2.31	3.87	0.81	0.22
GII.3	6.32	4.49	7.12	1.27
GII.4	3.36	4.82	5.24	9.08
GII.5	4.63	3.28	3.72	3.18
GII.6	0.63	0.65	2.62	1.85
GII.7	8.62	3.13	2.34	1.54
GII.8	1.86	6.25	6.78	1.59
GII.9	8.09	0.97	2.71	1.39
GII.10	1.75	6.81	5.59	1.54
GII.12	2.35	8.70	5.28	2.99
GII.13	3.20	4.92	4.58	0.26
GII.14	1.64	3.70	9.23	1.08
GII.15	0.00	8.84	0.88	5.09
GII.16	4.01	3.23	8.80	3.13
GII.17	0.82	3.28	9.62	0.24
GII.20	2.55	2.30	5.88	1.24
GII.21	1.61	2.40	1.52	1.33
GII.22	3.81	3.44	3.28	7.69

**TABLE 3:** Intra- and inter-assay coefficients of variation for the broad-spectrum detection of GII.4-5D41A12 monoclonal antibodies by ELISA.

GII.4-5D41A12	Intra-assay Precision CV/%			Inter-assay Precision CV/%
Antigen designation	1	2	3	–
GI.1	5.41	6.14	2.83	2.59
GI.2	1.81	2.41	1.32	8.92
GI.3	1.61	1.63	5.53	9.76
GI.4	2.71	4.27	2.34	8.71
GI.5	9.88	4.41	0.83	2.87
GI.6	4.20	0.00	6.40	4.07
GI.7	4.68	3.68	3.08	5.57
GI.8	0.00	6.83	3.63	8.63
GI.9	2.26	2.71	3.89	2.98
GII.1	0.00	1.82	2.79	1.23
GII.2	2.08	7.23	2.51	6.57
GII.3	8.52	3.76	1.95	8.95
GII.4	6.80	5.54	5.38	3.81
GII.5	3.12	1.68	1.79	3.26
GII.6	3.72	6.59	1.28	9.47
GII.7	4.27	2.36	1.79	2.28
GII.8	8.52	2.94	9.05	3.38
GII.9	0.98	1.76	1.64	5.77
GII.10	0.88	2.37	6.09	5.61
GII.12	1.67	2.11	5.62	3.61
GII.13	5.23	5.91	9.99	1.77
GII.14	8.65	4.13	5.95	0.30
GII.15	0.00	0.91	1.61	1.54
GII.16	2.44	2.30	5.00	2.85
GII.17	4.20	4.26	2.17	1.64
GII.20	2.22	2.31	3.74	5.82
GII.21	3.59	1.49	2.37	2.36
GII.22	4.68	0.83	5.01	2.52

**TABLE 4:** Intra- and inter-assay coefficients of variation for the broad-spectrum detection of GII.4 + GII.8 polyclonal antibodies by ELISA.

GII.4 + II.8	Intra-assay Precision CV/%			Inter-assay Precision CV/%
Antigen designation	1	2	3	–
GI.1	3.44	2.81	3.88	9.21
GI.2	5.49	3.50	4.21	0.94
GI.3	2.50	2.94	6.57	9.99
GI.4	3.26	4.40	2.81	1.27
GI.5	2.68	2.40	2.60	3.63
GI.6	6.46	4.00	5.03	4.48
GI.7	3.72	4.73	0.97	8.74
GI.8	3.77	1.27	1.24	9.16
GI.9	3.43	0.92	1.71	3.56
GII.1	4.67	1.43	3.76	4.39
GII.2	3.29	3.22	2.27	3.31
GII.3	4.35	4.22	3.07	1.88
GII.4	3.35	1.48	1.68	2.43
GII.5	4.03	6.63	5.23	3.83
GII.6	4.35	0.95	0.62	9.79
GII.7	4.59	1.88	3.77	2.50
GII.8	1.75	2.09	3.40	4.95
GII.9	4.46	0.55	5.55	2.25
GII.10	3.20	2.84	3.00	1.12
GII.12	0.82	1.10	1.01	2.25
GII.13	4.42	3.09	2.85	9.25
GII.14	3.65	1.58	2.52	2.73
GII.15	8.80	8.86	4.57	7.19
GII.16	2.43	3.79	4.28	2.12
GII.17	0.75	4.23	3.17	1.79
GII.20	1.76	0.81	4.09	1.89
GII.21	1.47	1.71	4.89	0.21
GII.22	2.03	0.49	6.46	3.27

## Discussion

Owing to the high frequencies of NoV outbreaks (Hall et al., 2011; Verhoef et al., 2013; Grytdal et al., 2016) and severe acute gastroenteritis (Atmar and Mullen, 2013; Cardemil et al., 2017), high risk of infection transmission (Sukhrie et al., 2010; Teunis et al., 2010; Verhoef et al., 2015), and enormous socioeconomic burden after infection (Pires et al., 2015), the development of NoV vaccines is critical for improving public health. However, the characteristics of NoV genetic diversity and high variation have markedly hindered vaccine development.

At present, NoV vaccines rely on VLP and P particles prepared by bioengineering technology as subunit vaccines. The morphology and antigenicity of recombinant NoV VLP are similar to natural virus particles (Estes et al., 2000). Preclinical studies have shown that VLP is immunogenic when administered by intranasal, oral or parenteral routes, which can induce serum and mucosal immune responses, and co-administration with mucosal adjuvants induces stronger immune responses (Estes et al., 2000). Three surface loops per P domain are potential sites for foreign antigen presentation, which could enhance the antigenicity and immunogenicity of antigens (Tan et al., 2011). Su et al. (2015) have shown that immune P particles induced antiserum in both mice (1:245,600) and rabbits (1:145,700), and also induced neutralizing antibodies to block the binding of NoV variants and receptors. The results prove the immunogenicity of P particles. Therefore, both NoV VLP and P particles are suitable choices for vaccine preparation.

Several NoV vaccines using a variety of different technologies have been developed, including three in clinical trials. The first is the bivalent intramuscular GI/GII.4 vaccine developed by Takeda Pharmaceutical Company Limited (Treanor et al., 2014). The second, developed by Vaxart, Inc., is based on a G I.1 NoV sequence (Kim et al., 2018). The third is the NoV tetravalent vaccine developed by Institut Pasteur of Shanghai Chinese Academy of Sciences and Chongqing Zhifei Biological Products Co., Ltd. (XINHUANET, 2019). Therefore, analysis of cross-protection among different strains is a significant step for the development of a successful NoV vaccine.

The immune cross-protection between different genotypes is complicated. Studies have shown GI.1 can induce blockade antibodies against other GI strains (Lindesmith et al., 2015); GII.4 antiserum and GII.17 antiserum have limited cross-reactivity or no cross-protection; Malm et al. (2015) demonstrated there is no cross-cluster protection between GI and GII, but the research of Czakó et al. (2015) showed GI.1 infection will cause an increase of GII.4 antiserum. In short, the development of effective vaccines must contain both GI and GII viruses.

In addition to vaccine development, detection technology is also an important part of NoV prevention and control. The immunoassay methods mainly include enzyme linked immunosorbent assay (ELISA) and immunochromatographic (ICG). In this study, capsid P proteins of 28 representative NoV strains were used as coating antigens for the development of an indirect ELISA to detect their binding ability with antibodies. The broad spectrum of NoV antisera could be quickly detected using this method. Furthermore, one genotype or a combination of genotypes with broad-spectrum detection could be screened for the design of multivalent vaccines. Owing to the genetic diversity of NoV and 5%–10% cross-reactivity observed among genotypes (lower cross-reactivity among genogroups), the development of a broad-spectrum multivalent vaccine platform is necessary to protect against as many NoV-prevalent strains as possible (LoBue et al., 2006).

In summary, in this study, we developed an indirect ELISA with the potential to serve as a rapid, reliable, and high-throughput method for screening broad-spectrum antibodies to expedite the development of NoV vaccines.

## Data Availability Statement

The original contributions presented in the study are included in the article/Supplementary Material, further inquiries can be directed to the corresponding authors.

## Author Contributions

YZ and LX designed the experiments and wrote the manuscript. YJ and WC contributed reagents, materials, and analysis tools. JG, YyL, and YhL performed the experiments. YLi, TC, and LW analyzed the data. MC and JW revised the manuscript. YD, JZ, and QW provided project funding. All authors have read and agreed to the published version of the manuscript.

## Conflict of Interest

The authors declare that the research was conducted in the absence of any commercial or financial relationships that could be construed as a potential conflict of interest. The handling editor declared a shared affiliation, with one of the author YD at the time of the review.

## Publisher’s Note

All claims expressed in this article are solely those of the authors and do not necessarily represent those of their affiliated organizations, or those of the publisher, the editors and the reviewers. Any product that may be evaluated in this article, or claim that may be made by its manufacturer, is not guaranteed or endorsed by the publisher.
