# Supplementary material for: Development and Application of a Novel Rapid and Throughput Method for Broad-Spectrum Anti-Foodborne Norovirus Antibody Testing
Source: Front Microbiol. 2021 Sep 3;12:670488. doi: 10.3389/fmicb.2021.670488 (PMC8446669; doi:10.3389/fmicb.2021.670488)
Supplement: Supplementary file 1 [file Data_Sheet_1.docx]

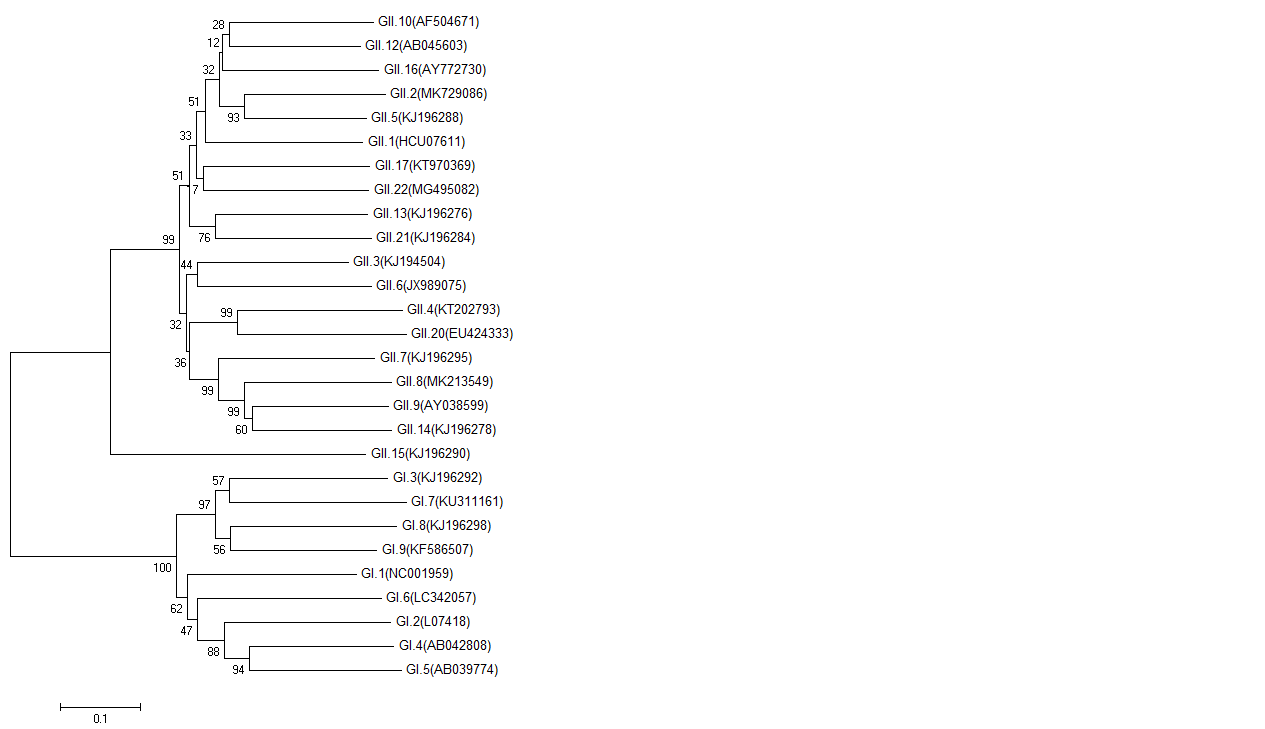


**Figure S1｜** Phylogenetic analyses based on the amino acid sequence of the P domain of 28 NoV strains. The maximum likelihood tree was constructed using MEGA (version 6.0). The branch significance was analyzed by bootstrap with 1000 replicates. The scale bar represents the unit for the expected number of substitutions per site.
